# Resting-state EEG reveals global network deficiency in prelingually deaf children with late cochlear implantation

**DOI:** 10.3389/fped.2022.909069

**Published:** 2022-09-06

**Authors:** Kaiying Lai, Jiahao Liu, Junbo Wang, Yiqing Zheng, Maojin Liang, Suiping Wang

**Affiliations:** ^1^Philosophy and Social Science Laboratory of Reading and Development in Children and Adolescents (South China Normal University), Ministry of Education, Guangzhou, China; ^2^Department of Otolaryngology, Sun Yat-sen Memorial Hospital, Guangzhou, China

**Keywords:** cochlear implantation, minimum spanning tree, implantation age, brain plasticity, functional networks, resting-state electroencephalography (EEG)

## Abstract

There are individual differences in rehabilitation after cochlear implantation that can be explained by brain plasticity. However, from the perspective of brain networks, the effect of implantation age on brain plasticity is unclear. The present study investigated electroencephalography functional networks in the resting state, including eyes-closed and eyes-open conditions, in 31 children with early cochlear implantation, 24 children with late cochlear implantation, and 29 children with normal hearing. Resting-state functional connectivity was measured with phase lag index, and we investigated the connectivity between the sensory regions for each frequency band. Network topology was examined using minimum spanning tree to obtain the network backbone characteristics. The results showed stronger connectivity between auditory and visual regions but reduced global network efficiency in children with late cochlear implantation in the theta and alpha bands. Significant correlations were observed between functional backbone characteristics and speech perception scores in children with cochlear implantation. Collectively, these results reveal an important effect of implantation age on the extent of brain plasticity from a network perspective and indicate that characteristics of the brain network can reflect the extent of rehabilitation of children with cochlear implantation.

## 1. Introduction

The cochlear implant (CI) is used worldwide as a rehabilitation method for children and adults with severe hearing loss (1, 2). However, the clinical outcomes of CI users, especially in terms of behavioral indicators such as speech perception, tend to vary widely across individuals (3, 4). Previous studies have suggested that the extent of brain plasticity after CI reception may be an important factor in explaining these individual differences (5). As brain plasticity is particularly high during a certain period of development (6), whether the timing of auditory restoration is within this sensitive period is particularly important. In other words, the age of CI reception may be a key factor influencing the extent of brain plasticity in CI users. However, how implantation age affects the extent of brain plasticity remains unclear. The present study therefore clarifies the importance of implantation age with respect to brain plasticity and attempts to investigate possible correlations between brain plasticity and restoration after CI reception.

Brain plasticity (BP) is an intrinsic, general and important property of the brain (7), which researchers have defined as changes or reorganizations in the brain during learning or interactions with the environment (8, 9); these changes in the brain include structural or functional reorganization (9, 10). Thus, BP changes in the brain following the absence of a sensory input and the subsequent provision of the input (11). The auditory cortex of prelingually deaf children with CIs constitutes a good model for the study of human BP mechanisms, because it enables comparison of these two conditions (12–15). Studies of deaf people and deaf animals have shown that there is considerable plasticity in the brain if hearing restoration takes place early in life (16, 17). By contrast, hearing acquisition later in life has less impact on BP (18). This means that there may be a sensitive period of BP (6, 19). For example, in normal-hearing (NH) individuals, synaptic density in the temporal lobe peaks at around 3.5 years old and then decreases (6, 20). Developmental abnormalities in synaptic plasticity lead to abnormal connectivity and functional disintegration (6, 16, 21), which can affect aspects of behavioral performance such as the development of speech perception. Therefore, providing children with CIs within the brief sensitive period allows for adequate cortical development (6). Also, restoring hearing early in life means that children with CIs are able to receive more feedback in their interactions with the environment (22, 23); such interactions are important in shaping the cognitive function of the brain. For example, prelingually deaf individuals developed speech skills if they received implants early in life (24, 25). Conversely, late implantees had less favorable outcomes (24, 25), usually manifesting as poor language comprehension (26). Therefore, providing CIs within the sensitive period is significant for postoperative restoration.

Previous studies have mainly measured the BP in specific regions (e.g., the auditory cortex) of CI users by manipulating experimental stimuli or tasks. Studies have consistently found that visual stimuli activated the auditory cortex, and the auditory cortex activation induced by visual information was related to language processing (27–32). Although these studies provided valuable evidence for the BP of CI users, there were several potential problems. First, in terms of task selection, some studies have reported that different stimuli could lead to differences in brain activation patterns in CI users. For example, lip-reading stimuli activated the left hemisphere in CI users (13), whereas checkboard stimuli activated the right hemisphere (30). These conflicting results suggest that the understanding of BP in CI users may be biased under different experimental tasks. In addition, as the age of CI users decreases, it has become necessary to consider using non-task methods appropriate for children with CIs to conduct relevant research. Moreover, in terms of analytical method, most previous studies have focused only on the activation of a specific region instead of paying sufficient attention to the relationship between functional brain regions and the characteristics of the brain network (33, 34). As the brain is a structurally and functionally complex network (34–37), considering it as a whole allows for a better understanding of BP. Finally, with regard to the study population, on the one hand, previous studies have mainly focused on post-lingually deaf adults with CIs (28, 30), who have acquired spoken language skills before the onset of hearing loss (38). However, it has been reported that these language experiences play an important part in the improvement of postoperative speech ability of CI users (39). Thus, it is not easy to strip away the factor of language experience when discussing BP in this context. On the other hand, owing to the sensitive period of BP, adults have poorer cortical plasticity compared with children (6). Therefore, the selection of adult subjects in previous studies is not conducive to exploring the potential contributions of BP in CI users to postoperative rehabilitation or speech improvement. For these reasons, selecting prelingually deaf children who received CIs within or outside the sensitive period may allow us to observe the contribution of implantation age to BP within the sensitive period, which will provide insight to guide clinical treatments.

Task-induced neural activity can often provide evidence of BP in CI users. However, as these CI users are usually very young, specific tasks may not be appropriate for them, which means that it is not easy to carry out relevant research. Fortunately, previous studies have shown that in addition to task-evoked neural activity, spontaneous neural activity in the resting state can also reveal intrinsic brain functional structures that reflect behavioral patterns (40–43). Furthermore, resting-state studies do not require responses from subjects; therefore, the resting state is widely used in studies of children with cognitive or developmental disabilities (44–46).

In electroencephalography (EEG) studies, the acquisition of resting-state data typically includes eyes-closed (EC) and eyes-open (EO) conditions. These two conditions are thought to reflect different states of brain activity (47, 48). Specifically, the EO condition is generally considered to reflect exteroceptive awareness, which is usually associated with attention and eye movements, whereas the EC condition usually reflects interoceptive awareness, particularly in relation to multisensory activity and imagination (49). In addition, activities of different frequency bands in resting-state EEG are associated with certain cognitive abilities or cognitive processes. For example, neural oscillations in the theta frequency band are associated with speech processing (50) and memory processing (51). There is further evidence that EEG resting-state activity could be used to characterize the topology of brain networks (52–54). Thus, studies of EEG resting-state data offer the possibility of exploring complex brain networks.

A brain network usually consists of a set of nodes (i.e., functional areas of the brain: auditory cortex, visual cortex, and somatosensory cortex) and links (i.e., connectivity between functional areas of the brain) (35, 55). Although classical brain network analysis can extract metrics describing network characteristics, it is difficult to avoid biased results when making comparisons across groups. This is because classical network analyses are susceptible to the effects of the density of individual brain networks (56). Brain network density refers to the number of potential connections in a brain network, which directly affects the metrics used to describe brain networks, such as the average path length that measures the degree of brain network integration (57). Therefore, in order to avoid biased results when comparing brain networks of the normally developing population with those of a sensory-impaired population, it is necessary to find a method that is independent of network density (56, 58). The minimum spanning tree (MST) approach provides an effective solution to this problem. MST analysis aims to build a brain network containing all nodes without any recurring cycles (57). Thus, the number of connections in the brain network are equal even for individuals from different groups, which avoids biasing of results due to differences in brain network connection density (57, 58).

There is accumulating evidence that MST analysis can capture subtle changes in brain networks during human development (59, 60), and this approach has been used extensively in EEG resting-state data from diverse populations, including people with deafness (61), autism (62), Alzheimer's disease (63), and dyslexia (46). In a recent EEG resting-state study, MST analysis was used to compare brain network characteristics between deaf and hearing controls in different resting states (61). The results showed differences in the topological characteristics of brain networks between the deaf group and the hearing group. Specifically, reduced global efficiency of brain networks was observed in the deaf group. These results suggest that early auditory deprivation may lead to abnormal brain network topological characteristics. Further analysis revealed that the number of years spent on sign language learning was related to characteristics of the brain network, that is, the longer the time spent on sign language learning, the higher the global brain efficiency (61). Although years of sign language learning were not directly representative of language processing ability, these findings indirectly suggested that changes in individual language levels could be reflected in network characteristics. For these reasons, the present study used a similar approach to directly compare the brain network characteristics of children who received CIs within the sensitive period, those who received CIs outside the sensitive period, and children with NH. We expected that brain network characteristics would change with the input of auditory stimuli after receiving CIs. Moreover, the changes in brain network characteristics would be expected to be different for CI receivers within and outside the sensitive period. We also explored the relationship between brain network characteristics and speech perception rehabilitation.

In the present study, EEG resting state data were collected from children with early CI reception (eCI) (age of implantation <3.5 years old), children with late CI reception (lCI) (age of implantation > 3.5 years), and NH children in both EO and EC conditions. Phase lag index (PLI) and MST analysis were used to calculate connectivity strength and brain network topology, respectively, and we compared the differences in these metrics among the three groups in different resting states. Finally, the MST metrics were correlated with speech perception scores. These analyses attempted to answer the questions of how BP is reflected in network characteristics in eCI and lCI children, and how brain network characteristics are related to speech rehabilitation outcomes of CI users. Specifically, we focused on the following aspects.(1) The effects of implantation age on the resting-state functional connectivity of brain regions. We examined the differences among groups in terms of the PLI values of auditory and visual regions, and auditory and parietal regions in two resting states, respectively.(2) The effects of implantation age on topological characteristics of brain networks. We investigated the differences among groups in the metrics describing brain networks in different resting states, which reflect the efficiency of information transfer. (3) The relationships between brain network metrics and speech perception scores. (4) Whether brain network characteristics of three groups differed under the two resting-state conditions.

To summarize, if implantation age had an effect on the extent of BP, then we would expect to observe that the connectivity patterns and network characteristics of the eCI children were similar to those of NH children; if not, there would be no difference in brain network characteristics between eCI children and lCI children. Finally, if speech perception scores were correlated with network characteristics, then the metrics derived from brain networks using MST analysis could reflect rehabilitation.

## 2. Methods

### 2.1. Participants

Fifty-five prelingually deafened Mandarin-speaking children with CIs who had sensorineural hearing loss were recruited. Their implants were on the right side. Among them, 31 had received their implants before 3.5 years old, whereas the other 24 had received their implants after 3.5 years old. Hence, these children were divided into two groups: eCI group (age of implantation <3.5 years old) and lCI group (age of implantation >3.5 years old). Another 29 Mandarin-speaking children with no history of hearing loss or neurological disorders were recruited as the NH group. Table 1 shows the demographic information of all participants. Nonverbal IQ scores were measured using Raven Standard Progressive Matrices Test (64), and there were significant differences among the three groups in IQ scores [*F*_(2, 81)_ = 4.876, *p* = 0.010, η^2^ = 0.107]. The NH group had higher IQ scores than the lCI group (*p* = 0.008), but there was no significant difference between the NH group and eCI group (*p* = 0.672), or the eCI group and lCI group (*p* = 0.150). There was no significant difference in duration of rehabilitation between the eCI and lCI groups [*F*_(1, 53)_ = 2.468, *p* = 0.122, η^2^ = 0.044]. There were significant differences in age between the eCI and lCI groups [*F*_(1, 81)_ = 4.554, *p* = 0.013, η^2^ = 0.101, NH: 9.1 ± 1.7 years old; eCI:8.8 ± 2.9 years old; lCI: 10.8 ± 2.9 years old], the lCI group was older than the eCI group (*p* = 0.017), and the lCI group was marginally older than the NH group (*p* = 0.054). Nonverbal IQ and age were used as the covariates for further analysis. Written parental informed consent was obtained for all the participants. All participants were recruited and tested in accordance with the Human Research Ethics Committee for Non-Clinical Faculties of South China Normal University (reference no. 158) and the Ethics Committee of Sun Yat-sen Memorial Hospital of Sun Yat-sen University.

**Table 1:** Demographical data for children with early cochlear implant (eCI), children with late cochlear implant (lCI) and children with normal hearing (NH).

	**NH group**					**eCI group**					**lCI group**					
	***n***	**M**	**SD**	**Range**		***n***	**M**	**SD**	**Range**		***n***	**M**	**SD**	**Range**		**F/t**
Implantation age	/	/	/	/		31	1.9	0.7	0.6–3.0		24	4.8	2.6	3.5–13.0		35.769***
Duration of rehabilitation	/	/	/	/		31	7.0	2.7	4.1–13.9		24	6.0	1.7	4.4–9.5		2.468
Age	29	9.1	1.7	6.0–13.9		31	8.8	2.9	5.9–16.0		24	10.8	2.9	7.6–18.8		4.827*
Nonverbal IQ	29	116	8.0	101–129		31	113	9.0	97–131		24	108	8.0	87–123		4.876*

^***^p < 0.01 and ^*^p < 0.05. NH group indicates normal hearing group; eCI group, early cochlear implant group; lCI group, late cochlear implant group.

### 2.2. Behavioral tests

Each participant completed speech perception tests including tone, vowel, and consonant discrimination. All materials were prepared in reference to Auditory Function Evaluation Criteria and Methods (65). All sound stimuli were recorded by a male native Mandarin-speaker with the Neundo 4 software. The sound stimuli were mono with a sampling rate of 44.1 kHz and a resolution of 1.6 bit. The Praat and the Sound Forge software were used to normalize the fundamental frequency, duration, and intensity of all sounds. The duration of stimuli was 500 ms with sound intensity of 75 dB SPL. The sound intensity of stimuli was measured before the experiment began; we stood 60 cm away from the loudspeakers and used a sound level meter to measure the sound intensity. The sound intensity of stimuli remained at 75 dB SPL. In addition, the experiment was carried out in a shielded room to prevent interference from noise. In each trial, children first heard a sound for 500 ms, then a blank screen would appear on the screen for 300 ms, before they were finally presented with another sound for 500 ms. Children were required to decide whether two consecutive sounds were the same as accurately and rapidly as possible.

### 2.3. EEG recording

EEG recordings took place in a dimly lit and sound-proofed room. Participants were seated at a distance of approximately 60 cm from the computer screen. Five min of EO resting-state and 5 min EC resting-state EEG data were recorded at a 1,000 Hz sampling rate using an EGI 128-channel dense array EEG system with the Hydrogel Geodesic Sensor Nets EGI 128-channel system (Electrical Geodesics Inc., USA). All children were seated in a chair where the height and seating position could be adjusted to give comfortable support. In the EO condition, participants were requested to focus on a black fixation on a gray background to minimize glare on the screen. The impedance of channels was kept below 40 *kΩ* during the recording. In previous studies using this EGI system, electrode impedances were kept below 50 or 60 *kΩ* (66, 67), so we considered the impedances of less than 40 *kΩ* to be acceptable in the present study.

## 3. Data analysis

### 3.1. Speech perception tests

We calculated the mean accuracy for the tone, vowel, and consonant perception tests. Analysis of variance (ANOVA) was used with mean accuracy as the dependent variable, with group (eCI, lCI, or NH) as a between-subject factor.

### 3.2. EEG preprocessing

The continuous EEG data were analyzed offline using EEGLAB 2019b, a MATLAB-based (MathWorks, Natick, MA, USA) open toolbox (68) for processing single-trial and/or averaged EEG data with any number of channels (68). The total duration of the raw EEG data was 5 min in both the EC condition and EO condition. EEG signals were digitally filtered with a band-pass of 0.5–30 Hz and resampled to 500 Hz, and eight channels associated with eye movements were deleted. The continuous EEG data were segmented into 2-s epochs. When removing artifacts, we treated subjects in the NH and CI groups differently; this was because for children with CIs, the EEG could be contaminated by electrical device-related artifacts (69). First, we visually inspected the epochs and removed those containing artifacts such as head or muscle movements, electrode cable movements, and rare jaw clenching. For children with CIs, a few electrodes (3–6) were removed near the CI to avoid poor separation of artifacts during independent component analysis (ICA) analysis (70). Subsequently, we performed an ICA and used automatic algorithm ADJUST (71) to identify independent components with artifacts. For the data from children with CIs, we also ran ICA and ADJUST repeatedly, a step designed to isolate and eliminate the artifacts associated with the CI. If the activated scalp showed a centroid on the side of the CI, then the independent component was defined as the CI artifact and removed (72). The remaining components were recalculated to create a filtered EEG dataset (72). Once this step of artifact rejection had been completed, we then interpolated the rejected electrodes. Furthermore, previous studies have found that using multiple epochs per subject increases the stability of network metrics (43, 61). Therefore, to ensure the stability of the results and to exclude the possible influence of the number of epochs per subject on the results, the number of epochs in the present study was set to 72 for all subjects in both the EC and EO conditions. Artifact-free data were re-referenced to the average of all scalp channels except the channels related to eye movement before obtaining functional connectivity and MST metrics. Both the PLI and MST metrics were calculated based on the 2-s epochs (72 epochs in total) for each subject. The EEG signals were band-pass-filtered into the delta (0.5–4 Hz), theta (4–8 Hz), alpha (8–13 Hz), and beta (13–30 Hz) frequency bands. Previous evidence suggests that the gamma frequency band in EEG recordings might be strongly influenced by muscle artifacts (45, 73), and this band has shown low reliability in graph analysis (66). Therefore, we excluded the gamma band in this study. Although the main purpose of the current study was not to examine the EEG power, referring to previous studies (45, 46), we performed EEG spectral power and relative power analyses; the results are reported in the Supplementary material.

### 3.3. Functional connectivity analysis

The phase lage index (PLI) was used to determine the functional connectivity between all pairs of 120 electrodes for each epoch and each frequency band. The PLI is a measure of the asymmetry of the distribution of instantaneous phase differences, determined using the Hilbert transformation, between two signals (74). The PLI is less affected by the montage and volume conduction when zero and π phase differences are ignored (61, 74). PLI values were obtained from the time series of phase differences Δϕ(*t*_*k*_) by the following equation: *PLI* = | < *sign*[*sin*(Δϕ(*t*_*k*_))] > | , where “sign” indicates the signum function. The PLI ranges between 0 and 1. A PLI value of 0 indicates no coupling or coupling with a phase difference centered around zero (mod π), whereas a PLI value of 1 indicates perfect phase locking at a value of Δϕ different from zero (mod π) (45, 46). PLI values closer to zero indicate weaker phase locking, whereas those closer to one suggest stronger phase locking (74). In the PLI analysis in the present study, we constructed 120 × 120 weighted adjacency matrices for each epoch and each frequency band for subsequent use in the MST analysis.

**Figure 1:**
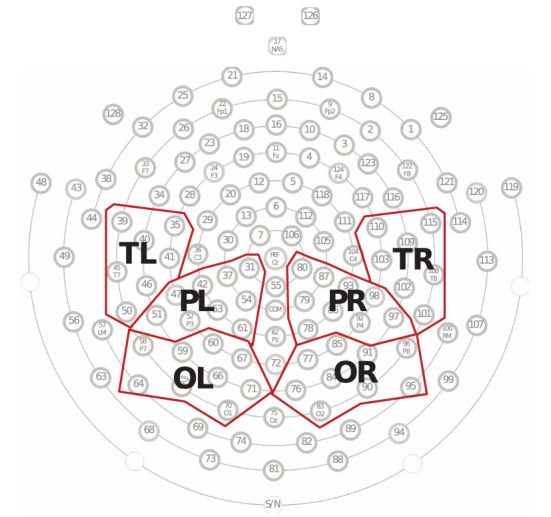
Electrode groupings. Fifty of 128 electrodes were divided into six regions, including the left temporal region (TL), right temporal region (TR), left parietal region (PL), right parietal region (PR), occipital region (OL), and right occipital region (OR). Each region contained between seven and nine electrodes, and the average of these electrodes represented the EEG responses for that scalp region.

In addition, we characterized the average PLI between the sets of electrodes in each group. For the analysis of connectivity strength, electrodes were divided into several regions of interests: left temporal region (TL; including the electrodes E35, E39, E40, E41, E45, E46, and E50, covering the left auditory cortex) (75); right temporal region (TR; including the electrodes E101, E102, E103, E108, E109, E110, and E115, covering the right auditory cortex) (75); left occipital region (OL; including the electrodes E58, E59, E60, E64, E65, E66, E67, E70, and E71, covering the left visual cortex) (76); right occipital region (OR; including the electrodes E76, E77, E83, E84, E85, E90, E91, E95, and E96, covering the right visual cortex) (76); left parietal region (PL; including electrodes E31, E37, E42, E47, E51, E52, E53, E54, and E61, covering the left somatosensory cortex) (76); and right parietal region (PR; including electrodes E78, E79, E80, E86, E87, E92, E93, E97, and E98, covering the right somatosensory cortex) (76) (see Figure 1).

### 3.4. MST analysis

A minimum spanning tree (MST) was constructed from the matrix containing the PLI values for each epoch. An MST is a unique subgraph based on a weighted matrix that connects all nodes (120 EEG electrodes were used in the present study) of the network without any loops or cycles (58). Thus, the MST contained 120 nodes (the number of nodes equaled the number of electrodes) and 119 links (the number of links was the number of *nodes*1). For each epoch, the MST was constructed using Kruskal algorithm (77), which has been widely used in previous studies (45, 61). The link weight was defined as 1/*PLI* in the present study. We first sorted the weights of all links in ascending order and selected the strongest-weighted link, then successively added the other stronger-weighted links until all nodes were connected by 119 links, eventually forming a subgraph without loops. Throughout the process, links were skipped if they formed a loop (58). Therefore, MST is the unique subgraph with the minimum total weights of all possible spanning trees.

**Figure 2:**
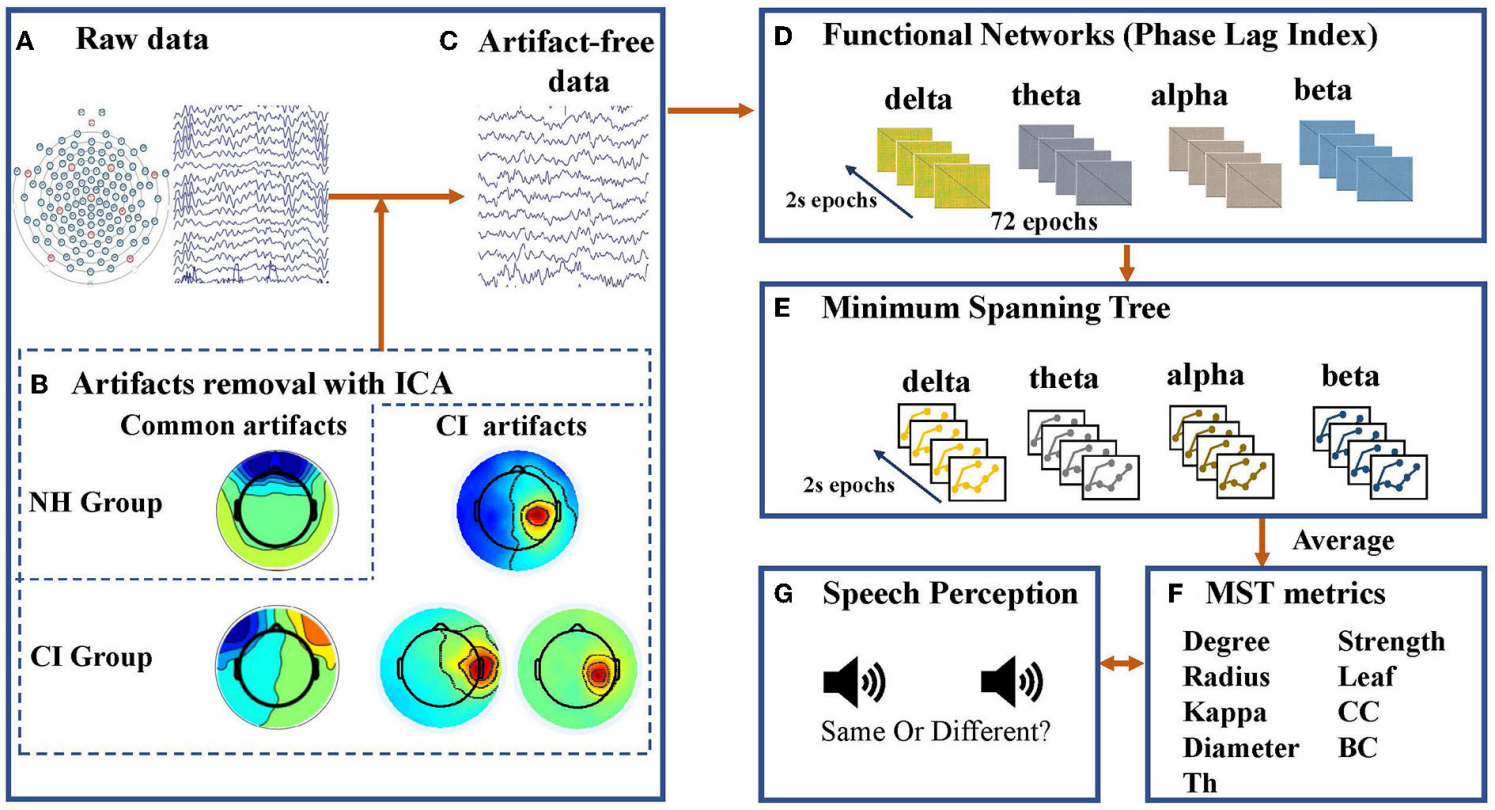
Schematic overview of minimum spanning tree (MST) analysis applied to electroencephalogram (EEG) data in this study. **(A)** Raw EEG data was collected by an EGI 128-channel dense array EEG system with Hydrogel Geodesic Sensor Nets. **(B)** Independent component analysis (ICA) was used to clean the epoched data. In addition to removing artifacts common to children with normal hearing (NH) and children with cochlear implants (CI), ICA was performed repeatedly on data from the CI group to isolate and eliminate the artifacts associated with the cochlear implant (CI artifacts). If the activated scalp showed a centroid on the side of the CI, then the independent component was defined as the CI artifact and removed. **(C)** Seventy-two artifact-free 2-s epochs were filtered for each frequency band (delta: 0.5–4 Hz, theta: 4–8 Hz, alpha: 8–13 Hz, and beta: 13–30 Hz). **(D)** A resting-state functional connectivity matrix based on the phase lag index (PLI) was constructed for each frequency band and each epoch. **(E)** Kruskal's algorithm was used to construct a minimum spanning tree (MST) matrix. **(F)** Topological characteristics of the tree can be characterized by metrics including maximum node degree (degree), leaf number (leaf), diameter, eccentricity, radius, strength, maximum betweenness centrality (BC), closeness centrality (CC), kappa, and tree hierarchy (Th). **(G)** Speech perception tests including tone, vowel, and consonant discrimination were complected by participants, and they were required to decide whether two consecutive sounds were the same as accurately and rapidly as possible.

MST analysis provides information about the topological characteristics of the tree (57, 58), which can be characterized by the following metrics: maximum node degree (degree), leaf number (leaf), diameter, eccentricity, radius, strength, maximum betweenness centrality (BC), closeness centrality (CC), kappa and tree hierarchy (Th). These metrics have been widely used in previous studies (45, 46, 57, 61). A detailed description of the MST metrics is presented in Table 2. Schematic overview of MST analysis in this study was shown in Figure 2.

**Table 2:** Graph metrics summary.

**Metric**	**Definition**
Maximum node degree	The node with the maximum number of connections
Leaf number	Nodes with degree = 1 in the MST
Diameter	Largest distance between any two nodes of the tree
Eccentricity	Longest distance between any reference and any other node
Radius	Smallest node eccentricity in the tree
Strength	A summation of all nodal connection weights
Maximum betweenness centrality	A network hub metric which relies on the identification of the number of shortest paths that pass through a node
Closeness centrality	Inverse of the sum of all distance to other nodes.
Kappa	Broadness of the degree distribution
Tree hierarchy	A hierarchical metric that quantifies the trade-off between large scale integration in the MST and the overload of central nodes

### 3.5. Statistical analysis

The PLI and MST metrics were log-transformed in an attempt to to obtain the statistical assumption of a normal distribution. To further verify that the data conformed to a normal distribution, the Shapiro-Wilk test was conducted separately for different metrics in each frequency band of each subject group. The results of the Shapiro-Wilk tests revealed that a few metrics did not conform to a normal distribution, including strength (mean), strength (maximum), and degree in the theta band; diameter and radius in the alpha band; and PLI values in the connection between right occipital region and right temporal region in the beta band. Therefore, for these non-normally distributed data, non-parametric repeated measures ANOVA was conducted using the Aligned Rank Transform Tool (ARTool) (78, 79), whereas for normally distributed data, parametric repeated measures ANOVA was used. For the PLI and MST metrics, parametric or non-parametric ANOVA was performed for the within-subjects factor (eye condition: EC and EO) and between-subjects factors (group: eCI, lCI, and NH). There were significant differences in age and IQ scores among the three groups; thus, age and IQ scores were used as covariates for statistical analysis. Homogeneity tests for parametric or non-parametric statistical analyses were carried out, and *p* > 0.05 was guaranteed. After obtaining the interaction results, we performed *post-hoc* tests with Bonferroni correction of *p*−values. Overall, the results obtained from the non-parametric analysis were similar to those from the parametric analysis, with the pattern of significant results remaining the same. Finally, correlation analyses were performed to determine any associations between behavioral scores and network metrics that differed among groups. Permutation tests were also performed on the correlations, and the Bonferroni-corrected *p*-values are reported.

## 4. Results

### 4.1. Speech perception tests

**Figure 3:**
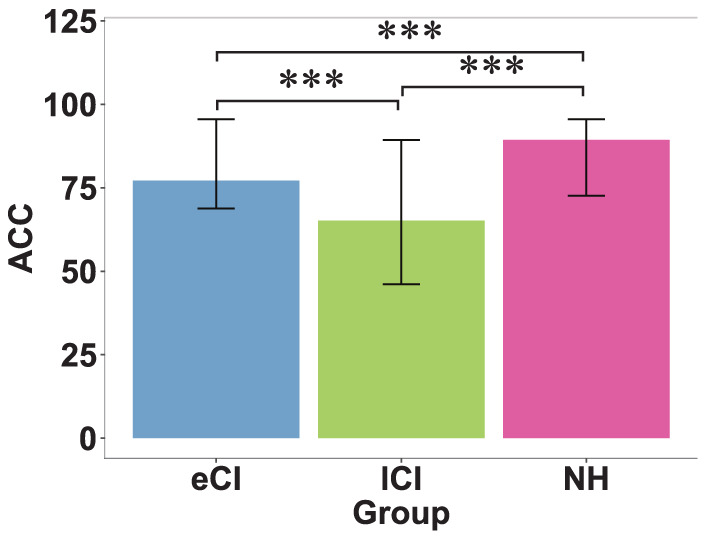
Behavioral results of speech perception tests. Accuracy (ACC) of speech perception in children with early cochlear implantation (eCI), children with late cochlear implantation (lCI), and children with normal hearing (NH) groups. Speech perception was measured as the mean accuracy for tone, vowel, and consonant discrimination. The NH group performed the best, followed by the eCI group, and the lCI group performed the worst. ^***^p < 0.001.

The results are reported in Table 3 based on completed tests (not all children completed the speech perception tests). A significant main effect of group on accuracy was found [*F*_(2, 69)_ = 52.69, *p* < 0.001, η^2^ = 0.604], with the NH group having higher scores than the eCI and lCI groups (*ps* < 0.001), and the eCI group having higher scores than the lCI group (*p* < 0.001) (see Figure 3).

**Table 3:** The results of speech perception test.

		**NH group**				**eCI group**				**lCI group**		

		***n***	**Mean**	**SD**		***n***	**Mean**	**SD**		***n***	**Mean**	**SD**
Speech perception	ACC (%)	24	89.37	5.13		29	77.16	6.8		21	65.16	10.56

### 4.2. Functional connectivity between sensory cortices

**Figure 4:**
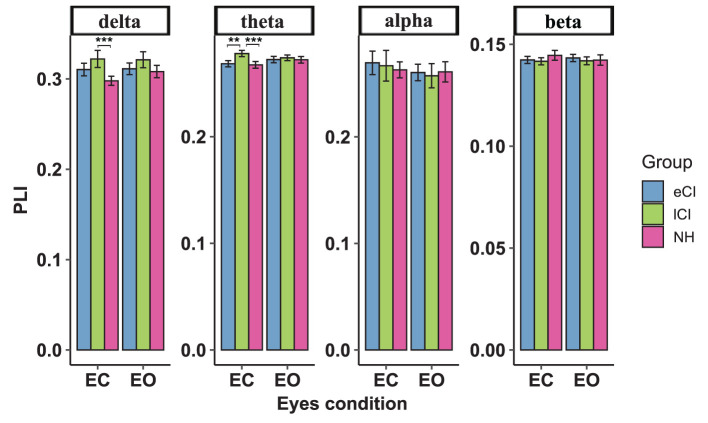
Functional connectivity between the left temporal region and left occipital region. The average functional connectivity between the left temporal region and left occipital region was defined by the phase lag index (PLI) (y-axis) under the eyes-closed (EC) and eyes-open (EO) conditions (x-axis) in the delta, theta, alpha, and beta frequency bands (top) in children with early cochlear implantation (eCI), children with late cochlear implantation (lCI), and children with normal hearing (NH). Interactions of eyes condition and group were found in delta and theta bands. In the delta band, lCI had higher PLI values than NH group, and in the theta band, lCI had higher PLI values than either eCI or NH children. ^**^p < 0.01 and ^***^p < 0.001.

**Figure 5:**
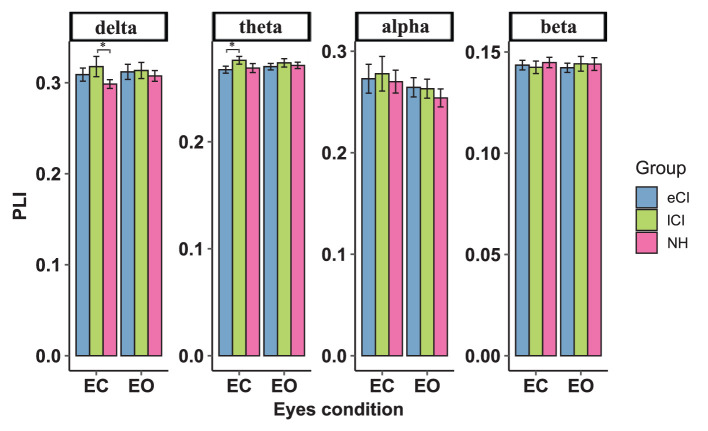
Functional connectivity between the right temporal region and right occipital region. The average functional connectivity between the right temporal region and right occipital region was defined by the phase lag index (PLI) (y-axis) under the eyes-closed (EC) and eyes-open (EO) conditions (x-axis) in the delta, theta, alpha, and beta frequency bands (top) in children with early cochlear implantation (eCI), children with late cochlear implantation (lCI) and children with normal hearing (NH). Interactions of eyes condition and group were found in the delta and theta bands. In the delta band, lCI children had higher PLI values than NH children, and in the theta band, lCI children had higher PLI value than either eCI or NH children. ^*^p < 0.05.

**Figure 6:**
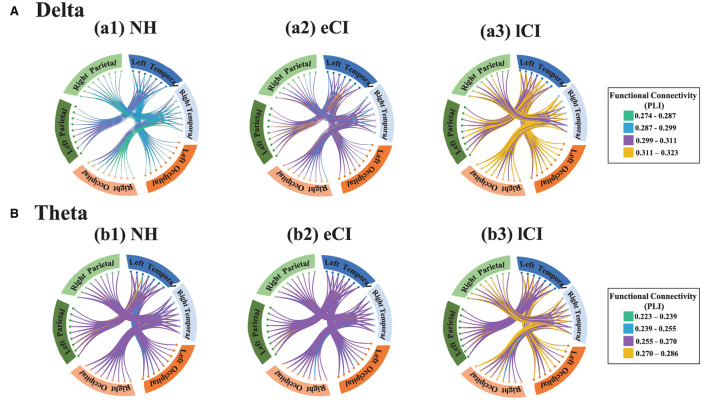
Functional connectivity between the temporal region and the occipital region and between the temporal region and parietal region in the delta band **(A)** and the theta band **(B)**. Small dots represent electrodes; dots of the same color belong to the same region, whereas those of different colors belong to different regions. The brain regions corresponding to each group of electrodes are shown around the circles: blue boxes represent the left temporal region, light blue boxes represent the right temporal region, orange boxes represent the left occipital regions, light orange boxes represent the right occipital region, dark green boxes represent the left parietal region, and light green boxes represent the right parietal region. The connecting lines between electrodes reflect phase lag index (PLI) values, i.e., the strength of the functional connectivity between regions, with green lines indicating the weakest connections and yellow ones indicating the strongest connections. In the delta band, the lCI group (a3) showed higher connection strength (more yellow connections) than the NH group (a1) or the eCI group (a2) between the temporal and occipital regions, both left and right. However, the connection strengths in the temporal and parietal regions were similar among the NH, eCI, and lCI groups in both the delta band and the theta band.

The functional connectivity strengths in the temporal and occipital cortex, temporal and parietal cortex for different frequency bands are shown in Figures 4–6. For connectivity between the left temporal and left occipital cortex, a significant interactions between condition and group were found in the delta band [*F*_(2, 79)_ = 4.076, *p* = 0.021, η^2^ = 0.094] and theta band [*F*_(2, 79)_ = 3.714, *p* = 0.029, η^2^ = 0.086]. Compared with the lCI group, the NH group showed weaker connectivity strength in the delta band (*p* < 0.001), whereas eCI and NH showed weaker strength in the theta band (*ps* < 0.003). For the right temporal and the right occipital cortex, marginally significant interactions were found in the delta band [*F*_(2, 79)_ = 2.985, *p* = 0.056, η^2^ = 0.070] and theta band [*F*_(2, 79)_ = 2.723, *p* = 0.072, η^2^ = 0.064]. Compared with the lCI group, NH showed weaker strength in the delta band (*p* = 0.011), and eCI showed weaker strength in the theta band (*p* = 0.011). There were no other statistically significant group differences in the temporal cortex and parietal cortex for any of the frequency bands (Supplementary Figures S2, S3).

### 4.3. MST metrics

**Figure 7:**
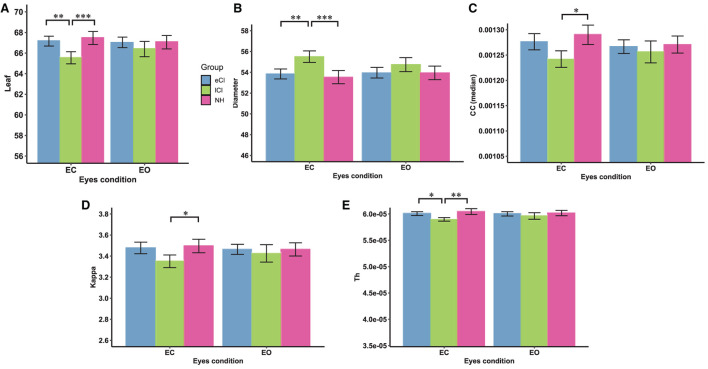
Comparison of network characteristics between the eyes-closed (EC) and eyes-open (EO) conditions for children with early cochlear implantation (eCI), children with late cochlear implantation (lCI), and children with normal hearing (NH) in the theta band (4–8 Hz). MST characteristics in the theta band are shown for the eCI group (blue), lCI group (green), and NH group (red) for both the EC and EO conditions (x-axis). The main differences between groups were found for **(A)** leaf number, **(B)** diameter, **(C)** closeness centrality (CC; median), **(D)** kappa, **(E)** tree hierarchy (Th). Compared with the lCI group, the NH and eCI groups had higher **(A)** leaf, **(D)** kappa, and **(E)** Th values and lower **(B)** diameters under the EC condition. ^*^p < 0.05, ^**^p < 0.01, and ^***^p < 0.001.

**Figure 8:**
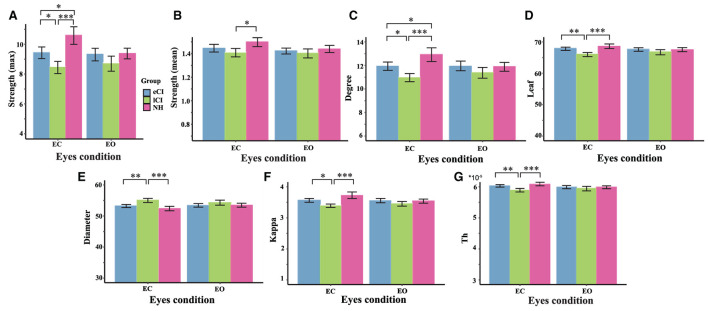
Comparison of network characteristics between the eyes-closed (EC) and eyes-open (EO) conditions in children with early cochlear implantation (eCI), children with late cochlear implantation (lCI), and children with normal hearing (NH) groups in the alpha band (8–13 Hz). MST characteristics in the alpha band are shown for the eCI group (blue), lCI group (green), and NH group (red) for both the EO and EC conditions (x-axis). The main group differences were found for **(A)** strength (max), **(B)** strength (mean), **(C)** degree, **(D)** leaf, **(E)** diameter, **(F)** kappa, and **(G)** tree hierarch (Th). ^*^p < 0.05, ^**^p < 0.01, and ^***^p < 0.001.

Some functional backbone characteristics differed among the three groups. MST analysis yielded significant interactions between group and condition in the theta (Figure 7) and alpha (Figure 8) bands; differences among the eCI, lCI, and NH groups were found only in the EC condition. For brevity, only significant differences are reported here; full details are given in Tables 4, 5.

**Table 4:** MST metrics on Delta and Theta bands of the children with early cochlear implantation (eCI), children with late cochlear implantation (lCI), and normal hearing children (NH) in eyes-open condition (EC) and eyes-closed condition (EO).

	**eCI**			**lCI**			**NH**	
	**EC**	**EO**		**EC**	**EO**		**EC**	**EO**
**Delta band**								
Strength (max)	10.252 ± 0.966	10.392 ± 1.279		9.763 ± 0.745	9.969 ± 1.076		9.824 ± 0.883	10.018 ± 0.843
Strength (mean)	1.650 ± 0.042	1.652 ± 0.041		1.645 ± 0.048	1.643 ± 0.039		1.643 ± 0.047	1.657 ± 0.053
Degree (max)	11.644 ± 1.026	11.793 ± 1.330		11.113 ± 0.901	11.350 ± 1.254		11.194 ± 1.012	11.342 ± 0.936
BC (max)	4,698 ± 65.519	4,698 ± 56.294		4,704 ± 44.024	4,703 ± 52.695		4,680 ± 50.093	4,701 ± 57.486
BC (median)	9.620 ± 6.397	11.229 ± 7.269		9.656 ± 5.891	11.880 ± 6.667		10.252 ± 6.634	14.726 ± 10.994
CC (max)	0.002 ± 1.1e-4	0.002 ± 8.3e-5		0.002 ± 9.8e-5	0.002 ± 8.36e-5		0.002 ± 1.2e-4	0.002 ± 1.4e-4
CC (median)	0.001 ± 6.1e-5	0.001 ± 4.9e-5		0.001 ± 7.2e-5	0.001 ± 5.3e-5		0.001 ± 5.8e-5	0.001 ± 8.0e-5
Leaf	66.837 ± 1.513	66.858 ± 1.768		65.836 ± 1.824	66.330 ± 2.117		66.077 ± 1.756	65.777 ± 1.631
Diameter	54.163 ± 1.513	54.142 ± 1.768		55.164 ± 1.824	54.670 ± 2.117		54.923 ± 1.756	55.223 ± 1.631
Eccentricity	15.685 ± 0.829	15.768 ± 0.708		15.852 ± 0.603	15.649 ± 0.513		15.924 ± 1.077	16.000 ± 1.166
Radius	10.419 ± 0.538	10.474 ± 0.448		10.544 ± 0.377	10.414 ± 0.345		10.590 ± 0.692	10.638 ± 0.762
Tree-hierarchy	6.0e-5 ± 1.1e-6	6.0e-5 ± 1.4e-6		5.9e-5 ± 1.4e-6	6.0e-5 ± 1.7e-6		6.0e-5 ± 1.5e-6	6.0e-5 ± 1.6e-6
Kappa	3.503 ± 0.172	3.535 ± 0.238		3.405 ± 0.158	3.455 ± 0.231		3.415 ± 0.161	3.433 ± 0.160
**Theta band**								
Strength (max)	9.465 ± 0.717	9.437 ± 0.630		8.992 ± 0.779	9.278 ± 0.969		9.419 ± 0.796	9.407 ± 0.778
Strength (mean)	1.530 ± 0.026	1.537 ± 0.021		1.550 ± 0.014	1.543 ± 0.023		1.528 ± 0.036	1.535 ± 0.020
Degree (max)	11.511 ± 0.918	11.427 ± 0.777		10.805 ± 0.949	11.197 ± 1.198		11.526 ± 1.041	11.389 ± 0.944
BC (max)	4,729 ± 62.275	4,724 ± 54.997		4,712 ± 67.432	4,717 ± 59.937		4,720 ± 59.017	4,714 ± 52.664
BC (median)	5.657 ± 3.273	7.058 ± 5.151		8.325 ± 4.492	9.321 ± 7.069		5.906 ± 3.852	7.573 ± 5.984
CC (max)	0.002 ± 7.6e-5	0.002 ± 6.2e-5		0.002 ± 7.4e-5	0.002 ± 8.9e-5		0.002 ± 8.2e-5	0.002 ± 7.7e-5
**CC (median)**	0.001 ± 4.5e-5	0.001 ± 3.8e-5		0.001 ± 4.0e-5	0.001 ± 5.3e-5		0.001 ± 5.2e-5	0.001 ± 4.5e-5
**Leaf**	67.162 ± 1.327	67.034 ± 1.422		65.539 ± 1.441	66.385 ± 1.820		67.462 ± 1.709	67.054 ± 1.755
**Diameter**	53.838 ± 1.327	53.966 ± 1.422		55.502 ± 1.362	54.740 ± 1.646		53.538 ± 1.709	53.946 ± 1.755
Eccentricity	15.115 ± 0.408	15.517 ± 0.372		15.408 ± 0.426	15.368 ± 0.438		15.105 ± 0.418	15.111 ± 0.440
Radius	10.078 ± 0.274	10.082 ± 0.251		10.287 ± 0.279	10.185 ± 0.371		10.067 ± 0.282	10.058 ± 0.286
**Tree-hierarchy**	6.0e-5 ± 9.9e-7	6.0e-5 ± 1.1e-6		5.9e-5 ± 8.2e-7	6.0e-5 ± 1.5e-6		6.0e-5 ± 1.5e-7	6.0e-5 ± 1.4e-6
**Kappa**	3.478 ± 0.152	3.464 ± 0.134		3.350 ± 0.147	3.425 ± 0.202		3.496 ± 0.172	3.463 ± 0.169

Bold text highlights significant results in group comparisons (*p* < 0.05).

**Table 5:** MST metrics on Alpha and Beta bands of the children with early cochlear implantation (eCI), children with late cochlear implantation (lCI) and normal hearing children (NH) in eyes-open condition (EC) and eyes-closed condition (EO).

	**eCI**			**lCI**			**NH**	
	**EC**	**EO**		**EC**	**EO**		**EC**	**EO**
**Alpha band**								
**Strength (max)**	9.429 ± 1.080	9.306 ± 1.182		8.439 ± 1.007	8.698 ± 1.237		10.583 ± 1.588	9.376 ± 0.962
**Strength (mean)**	1.447 ± 0.091	1.422 ± 0.069		1.408 ± 0.087	1.402 ± 0.093		1.500 ± 0.108	1.440 ± 0.081
**Degree (max)**	11.933 ± 0.988	11.958 ± 1.133		10.965 ± 0.839	11.370 ± 1.123		12.914 ± 1.578	11.882 ± 1.020
BC (max)	4,757 ± 59.022	4,774 ± 53.955		4,748 ± 54.910	4,752 ± 42.953		4,765 ± 65.028	4,776 ± 62.628
BC (median)	8.634 ± 4.617	8.122 ± 4.196		5.764 ± 3.890	5.820 ± 3.492		9.074 ± 4.840	8.736 ± 4.043
CC (max)	0.002 ± 1.8e-4	0.002 ± 1.2e-4		0.002 ± 1.6e-4	0.002 ± 1.7e-4		0.002 ± 2.2e-4	0.002 ± 1.6e-4
CC (median)	0.001 ± 1.1e-4	0.001 ± 7.5e-4		0.001 ± 9.5e-4	0.001 ± 1.1e-4		0.001 ± 1.3e-4	0.001 ± 9.6e-4
**Leaf**	67.791 ± 1.454	67.575 ± 1.614		65.979 ± 1.606	66.697 ± 2.005		68.605 ± 1.961	67.524 ± 1.707
**Diameter**	53.209 ± 1.454	53.425 ± 1.614		55.021 ± 1.606	54.303 ± 2.005		52.395 ± 1.961	53.476 ± 1.707
Eccentricity	14.828 ± 1.067	14.653 ± 0.543		15.139 ± 0.783	14.853 ± 0.622		14.772 ± 0.813	14.635 ± 0.474
Radius	9.886 ± 0.690	9.783 ± 0.347		10.106 ± 0.494	9.902 ± 0.401		9.854 ± 0.527	9.768 ± 0.312
**Tree-hierarchy**	6.0e-5 ± 1.1e-6	6.0e-5 ± 1.4e-6		5.9e-5 ± 1.4e-6	5.9e-5 ± 1.7e-6		6.1e-5 ± 1.5e-6	6.0e-5 ± 1.3e-6
**Kappa**	3.564 ± 0.173	3.556 ± 0.195		3.389 ± 0.134	3.452 ± 0.182		3.728 ± 0.285	3.538 ± 0.179
**Beta band**								
Strength (max)	4.842 ± 0.469	4.682 ± 0.408		4.647 ± 0.395	4.750 ± 0.461		4.793 ± 0.480	4.763 ± 0.612
Strength (mean)	0.833 ± 0.016	0.829 ± 0.020		0.836 ± 0.023	0.833 ± 0.030		0.841 ± 0.028	0.835 ± 0.024
Degree (max)	10.495 ± 0.990	10.199 ± 0.876		10.015 ± 0.751	10.301 ± 1.048		10.253 ± 0.779	10.264 ± 1.076
BC (max)	4,750 ± 44.573	4,750 ± 57.644		4,732 ± 65.966	4,757 ± 68.173		4,737 ± 54.528	4,760 ± 60.509
BC (median)	13.190 ± 9.150	13.005 ± 8.251		17.242 ± 9.876	17.003 ± 9.569		12.970 ± 9.626	14.072 ± 8.969
CC (max)	0.004 ± 1.5e-4	0.004 ± 1.6e-4		0.004 ± 1.6e-4	0.004 ± 2.8e-4		0.004 ± 1.3e-4	0.004 ± 1.6e-4
CC (median)	0.002 ± 8.7e-5	0.002 ± 9.5e-5		0.002 ± 9.1e-5	0.004 ± 1.6e-4		0.002 ± 8.3e-5	0.002 ± 9.4e-5
Leaf	65.291 ± 1.763	65.101 ± 1.794		64.514 ± 1.572	65.008 ± 2.150		65.278 ± 1.832	65.061 ± 2.102
Diameter	55.709 ± 1.763	55.899 ± 1.794		56.486 ± 1.572	55.992 ± 2.150		55.722 ± 1.832	55.939 ± 2.102
Eccentricity	14.697 ± 0.532	14.691 ± 0.508		14.786 ± 0.427	14.665 ± 0.608		14.598 ± 0.484	14.712 ± 0.503
Radius	9.813 ± 0.352	9.812 ± 0.335		9.874 ± 0.287	9.795 ± 0.398		9.772 ± 0.322	9.832 ± 0.337
Tree-hierarchy	5.8e-5 ± 1.4e-6	5.8e-5 ± 1.2e-6		5.8e-5 ± 1.3e-6	5.8e-5 ± 1.5e-6		5.8e-5 ± 1.6e-6	5.8e-5 ± 1.5e-6
Kappa	3.307 ± 0.154	3.273 ± 0.139		3.237 ± 0.123	3.278 ± 0.179		3.284 ± 0.137	3.279 ± 0.199

Bold text highlights significant results in group comparisons (*p* < 0.05).

In the theta band, there were significant interactions for leaf [*F*_(2, 79)_ = 5.070, *p* = 0.008, η^2^ = 0.114], diameter [*F*_(2, 79)_ = 4.163, *p* = 0.019, η^2^ = 0.095], kappa [*F*_(2, 79)_ = 3.607, *p* = 0.032, η^2^ = 0.084], Th [*F*_(2, 79)_ = 3.922, *p* = 0.024, η^2^ = 0.020], and CC median [*F*_(2, 79)_ = 3.765, *p* = 0.027, η^2^ = 0.087]; all differences among groups were in the EC condition. More specifically, compared with the lCI group, the NH and eCI groups had higher leaf (*ps* < 0.009), kappa (*ps* < 0.064), and Th (*ps* < 0.043) values and lower diameter (*ps* < 0.006). Moreover, the lCI group had a higher CC median than the eCI group (*ps* = 0.016).

In the alpha band, there were significant interactions for max strength [*F*_(2, 79)_ = 9.815, *p* < 0.001, η^2^ = 0.199], mean strength [*F*_(2, 79)_ = 3.04, *p* = 0.053, η^2^ = 0.072], degree [*F*_(2, 79)_ = 8.296, *p* < 0.001, η^2^ = 0.174], leaf [*F*_(2, 79)_ = 8.419, *p* < 0.001, η^2^ = 0.176], diameter [*F*_(2, 79)_ = 5.440, *p* = 0.006, η^2^ = 0.121], Th [*F*_(2, 79)_ = 6, 842, *p* = 0.002, η^2^ = 0.148], and kappa [*F*_(2, 79)_ = 10.127, *p* < 0.001, η^2^ = 0.204]; differences among groups were only found in the EC condition. More specifically, compared with the lCI group, the NH group had higher max strength (*p* < 0.001), mean strength (*p* = 0.027), degree (*p* < 0.001), leaf (*p* < 0.001), Th (*p* < 0.001), and kappa (*p* < 0.001). In addition, compared with the lCI group, the eCI group had higher max strength (*p* = 0.027), degree (*p* = 0.017), leaf (*p* = 0.002), Th (*p* = 0.009), and kappa (*p* = 0.016). The NH group had higher max strength (*p* = 0.028) and degree (*p* = 0.043) than the eCI group. The lCI group had larger diameter than the eCI (*p* = 0.003) and NH (*p* < 0.001) groups. Notably, in the NH group, differences between the EC and EO conditions were found (*ps* < 0.006). Compared with the EO condition, the EC condition in the NH group had larger max strength (*p* < 0.001), mean strength (*p* < 0.001), degree (*p* < 0.001), leaf (*p* = 0.006), kappa (*p* < 0.001), and Th (*p* = 0.005), and lower diameter (*p* = 0.004).

Finally, for the delta and beta bands, there were no significant interactions or main effects of group or condition.

### 4.4. MST metrics and speech perception scores

**Figure 9:**
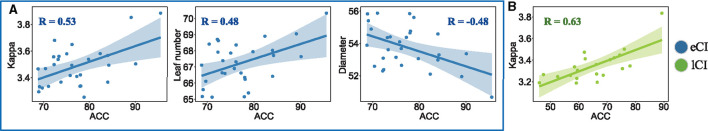
Correlations between accuracy (ACC) of speech perception and minimum spanning tree (MST) metrics in the theta frequency band (4–8 Hz) in **(A)** children with early cochlear implantation (eCI; blue markers) and **(B)** children with late cochlear implantation (lCI; green markers). In the eCI group, ACC of speech perception was significantly positively correlated with kappa (*r* = 0.53, *p* = 0.032) and leaf (*r* = 0.47, *p* = 0.048) and a negatively correlated with diameter (*r* = −0.47, *p* = 0.059). In the lCI group, ACC of speech perception was significantly positively correlated with kappa values (*r* = 0.63, *p* = 0.023). Permutation tests were performed randomly, assigning subjects to three groups 5,000 times, and Bonferroni-corrected p-values are reported.

Correlation analysis was performed to examine the associations between functional backbone characteristics in the EC condition and the accuracy of speech perception (ACC). Speech perception was related to MST characteristics in the theta band; the most pronounced effects were found in the CI groups (Figure 9). More specifically, ACC showed a significant positive correlation with kappa in both the eCI group (*r* = 0.53, *p* = 0.032) and lCI group (*r* = 0.63, *p* = 0.023), a significant positive correlation with leaf in the eCI group (*r* = 0.47, *p* = 0.048), and a trend of negative correlation with diameter in the eCI group (*r* = −0.47, *p* = 0.059). However, we did not find a significant correlation for the NH group (*ps* > 0.099) in any frequency band.

## 5. Discussion

The aim of the present study was to examine the effects of implantation age on the extent of BP from a brain network perspective using EEG resting states, and to further investigate the relationships between network characteristics and speech perception scores. Inspired by previous studies, PLI and MST analysis were used to calculate the resting-state functional connectivity and network topology characteristics, respectively. It was found that the connectivity between the occipital and temporal regions was stronger in lCI children than in eCI children and NH children. In addition, the lCI group had lower information transfer efficiency than the NH group, whereas the efficiency of the eCI group was similar to that of the NH group. However, the effect of group differences was only found in the EC condition. More importantly, correlations were found between some MST metrics and speech perception scores.

### 5.1. Implantation age affects brain network characteristics

The present study found enhanced resting-state functional connectivity between visual and auditory regions in lCI children compared with both eCI children and NH children. These results are similar to those of previous studies, in which new and stronger connectivity between visual and auditory areas was found in deaf animals (80–82), and also in deaf humans (83). These results support the finding of enhanced connectivity between auditory and visual areas in lCI children in this study. However, unlike some previous work, the present study found a connectivity effect on both sides of the brain, which suggested that the bilateral functional connectivity of the resting brain offered the possibility of processing different types of stimuli in children with CI (84).

However, no group differences were found in the resting-state functional connectivity of the temporal and parietal regions in this study. Previous studies have reported that enhanced functional connectivity between auditory and parietal regions might be related to sign language processing (85, 86); this could provide an explanation of our results, as all subjects in the present study lacked sign language experience.

More importantly, based on the results of MST analysis, differences were found between the characteristics of eCI children and those of lCI children in the EC condition. Moreover, lCI children showed reduced global efficiency compared with other groups. Specifically, higher diameter values and lower leaf, kappa, Th, and degree values were found in lCI children compared with eCI children and NH children. For each metric, a smaller diameter indicates lower connectivity strength between brain network nodes (34); a larger leaf value indicates more connections between a node and the rest of the network (35), and a smaller degree usually indicates less integration of the brain network (58). These results suggest that under the EC condition, brain networks in the lCI group were less integrated and their topology was more path-like (i.e., less integrated and with reduced global efficiency) (57). Previous studies have examined the corticocortical connectivity in congenitally deaf animals, and found reduced induced responses, indicating the reduced corticocortical connectivity (87). This suggests that auditory deprivation affects the global network integration; thus, the lCI group would have less integration and a more similar pattern to that of the deaf group owing to late implantation.

The results of the MST analysis also revealed that the brain network of eCI group had higher integration than that of the lCI group. Although some metrics in the early implantation group were lower than those in the NH group (e.g., degree in the alpha band), the overall results showed that the brain network characteristics of the eCI group were similar to those of the NH group. Subjects in our study were prelingually CI children, and there was no difference in duration of rehabilitation as we controlled the age factor. Therefore, it is reasonable to assume that the group effects stemmed from implantation age, which influenced the extent of BP, as reflected in the characteristics of the brain network.

These results demonstrate that auditory deprivation has a long lasting effect on brain networks, mainly resulting in reduced brain network efficiency in children with CI (61, 88, 89). However, it is clear from the results that when implantation occurs within the sensitive period, there is an opportunity for the brain network characteristics of CI children to be restored to the level of those of NH children. By contrast, children receiving CIs outside the sensitive period may be affected by auditory deprivation, i.e., residual preimplantation reorganization.

### 5.2. Differences in frequency bands reflect specificity of brain plasticity

Notably, the present study only found differences in brain network characteristics among groups in the theta and alpha frequency bands, suggesting that BP is not reflected in all frequency bands. We therefore hypothesized that the effects of auditory deprivation on BP would also be limited to specific frequency bands. A study found that the effects of auditory deprivation were only evident in the alpha and beta frequency bands (61). This might be related to the functions reflected by each frequency band (51, 90). For example, synchronous oscillatory activity in lower frequency bands (e.g., the alpha band) is associated with long-range interactions in top-down processing, such as working memory (91). Several studies have used the phase-based measures similar to those used in the present study to investigate cortical connectivity within the auditory cortex (92, 93), suggesting that the loss of alpha oscillations primarily affected top-down interactions in the auditory cortex. Compared to the (mid-range) auditory interareal connectivity found in previous studies, more theta effects were well consistent by the long-range synchronization in the present study (92, 93). Moreover, we found group effects in the theta band; activity in the theta frequency band has an important role in language processing (50, 94). The relationship between MST metrics and speech perception found in this study supports the previous suggestion that abnormalities in theta oscillations are associated with difficulties in speech perception (95, 96). A detailed description of the correlation results is provided in Section 5.3.

### 5.3. Less integrated network indicates weak speech perception ability

The results of speech perception tests showed that NH children performed better than those in the two CI groups. Furthermore, the eCI group performed better than the lCI group. These results suggest that implantation age has an important role, consistent with previous behavioral results (97–100).

Interestingly, correlations were found between MST metrics and speech perception in the theta band in children with CI. Specifically, speech perception scores were positively correlated with kappa and leaf number and negatively correlated with diameter; this suggests that in CI children, lower efficiency of brain networks was associated with worse speech perception scores. The integration of brain networks is often related to efficiency of information transfer (46). Deficits in neuronal and synaptic connections due to auditory deprivation may lead to less efficient information transfer (101), which is detrimental to language processing (46). However, the relationship between MST metrics and speech perception scores was only reflected in the CI children, not in the NH children. These results could be interpreted in two ways. On the one hand, the MST metrics may be an important measurement of speech perception ability in CI children. On the other hand, the relatively high homogeneity of the NH children compared with the CI children may explain the results.

However, the present study did not find any correlations in the alpha band, probably because neural oscillations in the alpha band are associated with working memory and visual attention (34). As auditory deprivation affects visual attention distribution (102, 103), the relationship between alpha neural oscillations and visual attention distribution could be studied in future work.

### 5.4. Lack of difference between EC and EO conditions in CI group

In the present study, differences between the EC and EO conditions were found only in the NH group, not in the eCI and lCI groups. In the NH group, degree, kappa, and Th in the alpha band were higher whereas diameter was lower in the EC condition than in the EO condition. These results are consistent with those of previous studies that found less integration in the EO condition and more integration in the EC condition (49, 52, 54, 104).

However, the present study did not find any differences in MST metrics between the EC and EO conditions in CI children. This is inconsistent with a previous study, where increased differences in functional network topology were found between the EC and EO conditions under the influence of auditory deprivation (61). We speculate that physiological age might account for these conflicting results; the mean age of deaf subjects selected in the previous study was about 18 years (61), whereas the mean age of CI children in this study was 9 years. In addition, compared with the EO condition, the EC condition provides more reliable measurements owing to the lack of interference from the external environment (e.g., visual stimuli) (49, 105) functional. This could also explain why the group effects on connectivity and brain characteristics found in this study only occurred in the EC condition.

### 5.5. Limitations and future directions

The present study had several limitations. First, there are different aspects of language processing, such as phonological processing, lexical processing, and sentence processing. However, this study only focused on tones, vowels, and consonants; it did not fully examine the language skills of CI children. Therefore, future studies need to investigate the associations between MST metrics and different elements of the language hierarchy to better understand the effects of implantation age. Second, although this study examined CI children speech perception, it did not explore how speech perception could be improved. In future studies, the effects of different training methods on speech perception could be investigated. Third, in the present study, we focused primarily on resting-state functional connectivity between the auditory and visual regions and neglected other potentially meaningful inter-regional connections. Therefore, to better understand the impact of age at implantation on brain connectivity, a comprehensive and careful analysis of inter-regional connectivity will be necessary in the future. Fourth, EEG network modularity is also a proxy of cognitive plasticity, and is considered to be a reliable neural marker of the development of language (67). This seems to suggest that modularity can reflect BP due to changes in sensory experience. The applications of modularity analysis of EEG data of hearing-impaired children could be investigated in future studies. Finally, this study compared the brain network characteristics of three groups and obtained valuable results, but it was not possible to understand the dynamic changes in brain network characteristics at an individual level. Future studies need to use longitudinal studies to gain insight into how brain network characteristics change as a result of auditory deprivation and subsequent use of CI.

## 6. Conclusion

Using resting-state EEG data and MST analysis, the present study investigated the effect of age of CI reception on BP, and the associations between brain network characteristics and speech perception scores. The results showed that the eCI group had higher brain network efficiency compared with the lCI group. The findings demonstrated the significance of implantation age in terms of various metrics derived from MST analysis. In addition, the correlations between network characteristics and speech perception scores suggest that brain network metrics can reflect the extent of rehabilitation after reception of CI.

## Data availability statement

The raw data supporting the conclusions of this article will be made available by the authors, without undue reservation.

## Ethics statement

The studies involving human participants were reviewed and approved by the Ethics Committee of Sun Yat-sen University and South China Normal University.

## Author contributions

KL and SW contributed to conception and design of the study and wrote the manuscript. KL, YZ, JW, and JL performed the experiments. KL and ML performed the data analyzes. SW, JL, and ML provided supervision and funding supports. All authors contributed to the article and approved the submitted version.

## Funding

This work was supported by the project of the National Natural Science Foundation of China (Code: 32171051, principal: SW) provides personnel and materials support and the publication fee, the project of the National Natural Science Foundation of China Youth Science Foundation (Code: 81800922, principal: ML) for instruments, and the Youth Science Foundation Project of the National Natural Science Foundation of China (Code: 81900954, personal in charge: JL) provides the support of personnel and sites.

## Conflict of interest

The authors declare that the research was conducted in the absence of any commercial or financial relationships that could be construed as a potential conflict of interest.

## Publisher's note

All claims expressed in this article are solely those of the authors and do not necessarily represent those of their affiliated organizations, or those of the publisher, the editors and the reviewers. Any product that may be evaluated in this article, or claim that may be made by its manufacturer, is not guaranteed or endorsed by the publisher.
