# Supplementary material for: Resting-state EEG reveals global network deficiency in prelingually deaf children with late cochlear implantation
Source: Front Pediatr. 2022 Sep 6;10:909069. doi: 10.3389/fped.2022.909069 (PMC9487891; doi:10.3389/fped.2022.909069)
Supplement: Supplementary file 1 [file Data_Sheet_1.pdf]

Supplementary Material

1 SPECTRAL POWER

1.1 Spectral Power analysis

We calculated the spectral power using fast fourier transformation (FFT) with a frequency resolution of $1/2s = 0.5$ Hz (Xue et al., 2020). The power spectra were averaged across all scalp electrodes. The power values were calculated for the following frequency bands: delta (0.5-4 Hz), theta (4-8 Hz), alpha (8-13Hz), and beta (13-30 Hz) (Fraga González et al., 2018). The relative power was calculated as the ratio of the power of the corresponding band to the total power. The main reason for examining the spectral power in the different frequency bands was to determine the attentional state of subjects and ensure the validity of the results of the present study.

1.2 Spectral Power and Relative Power

Spectral power plots were shown in Figure S1, and values of spectral power and relative power were shown in Table S1. No significant differences were found among the three groups with respect to individual alpha peak frequencies. We found no statistically significant differences among the three groups in the EC condition ($F_{(2,81)} = 0.407$, $p = 0.667$, $\eta^2 = 0.010$); the mean (SD) peak frequencies for the NH, eCI, and lCI groups were 9.32 (0.78), 9.11 (0.92), and 9.23 (1.08), respectively. We also found no significant differences among the three groups in the EO condition ($F_{(2,81)} = 0.709$, $p = 0.495$, $\eta^2 = 0.017$); the mean (SD) peak frequencies for the NH, eCI, and lCI groups were 9.22 (0.88), 8.98 (0.84) and 9.23 (1.10), respectively.
For spectral power, we found a significant interaction between group and eye condition only in the alpha band ($F_{(2,79)} = 4.701$, $p = 0.012$, $\eta^2 = 0.106$). In the EC condition, the NH group showed higher power than the lCI group ($p = 0.012$), and the eCI group showed marginally higher power than the lCI group ($p = 0.074$), but no similar results were found in the EO condition. No significant interaction in any other bands was found.
For the relative power, we found a significant interaction between group and eye condition in the alpha band ($F_{(2,79)} = 9.680$, $p < 0.001$, $\eta^2 = 0.197$), with the lCI group showing lower power than the NH group ($p = 0.002$) and the eCI group ($p = 0.061$) in the EC condition, but no similar results were found in the EO condition. Moreover, we found a significant interaction between group and eye condition in the delta band ($F_{(2,79)} = 3.640$, $p = 0.031$, $\eta^2 = 0.084$), with the lCI group showing higher delta relative power than the NH group ($p = 0.012$).

REFERENCES

Fraga González, G., Smit, D. J., Van der Molen, M. J., Tijms, J., Stam, C. J., De Geus, E. J., et al. (2018). Eeg resting state functional connectivity in adult dyslexics using phase lag index and graph analysis. *Frontiers in human neuroscience* 12, 341
Xue, H., Wang, Z., Tan, Y., Yang, H., Fu, W., Xue, L., et al. (2020). Resting-state eeg reveals global network deficiency in dyslexic children. *Neuropsychologia* 138, 107343

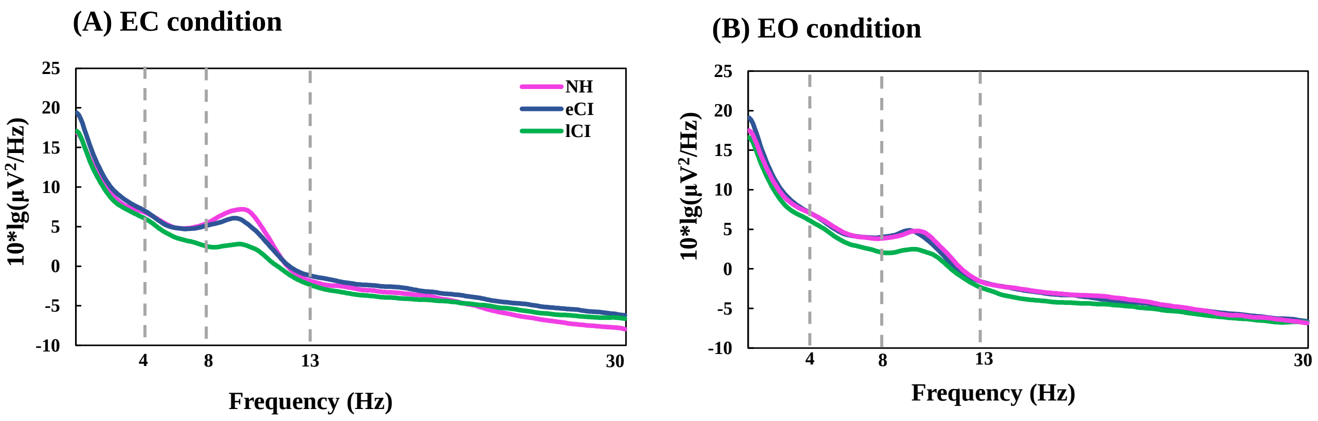

Figure S1. Fast fourier transformation (FFT) power spectra averaged across all scalp electrodes in the (A) eye-closed (EC) and (B) eyes-open (EC) conditions. The vertical dashed lines indicate the boundaries of the main frequency bands (4, 8, 13, and 30 Hz). The red line indicates the data from the normal hearing group (NH), the blue line indicates data of children with early cochlear implantation (eCI), and the green line indicates data of the children with late cochlear implantation (lCI).In the EC condition, the NH and eCI groups showed higher power than the lCI group, but there were no between-group differences in the EO condition.

Table S1. Spectral power ($10 * lg(\mu V^2)$) and relative power of delta, theta, alpha, and beta frequency bands in children with normal hearing (NH), children with early cochlear implants (eCI), and children with late cochlear implants (lCI).

	NH		eCI		lCI	
	EC	EO	EC	EO	EC	EO
Spectral Power						
Delta	10.48±2.73	10.59±2.60	11.73±3.19	11.21±3.31	9.83±3.42	9.57±3.18
Theta	5.45±3.09	4.61±2.73	5.43±3.01	4.55±3.01	3.63±2.74	3.23±2.61
Alpha	3.58±2.79	2.21±2.18	3.14±2.21	1.94±2.69	0.79±2.21	0.75±2.13
Beta	-4.99±1.95	-4.37±1.79	-3.83±1.92	-4.61±2.22	-4.86±1.71	-4.86±1.75
Relative Power						
Delta	0.57±0.11	0.64±0.11	0.61±0.11	0.64±0.11	0.69±0.14	0.69±0.14
Theta	0.16±0.05	0.14±0.04	0.14±0.04	0.13±0.03	0.13±0.05	0.12±0.05
Alpha	0.21±0.10	0.14±0.06	0.17±0.09	0.15±0.09	0.10±0.08	0.10±0.08
Beta	0.07±0.03	0.08±0.03	0.08±0.03	0.08±0.03	0.08±0.05	0.08±0.05

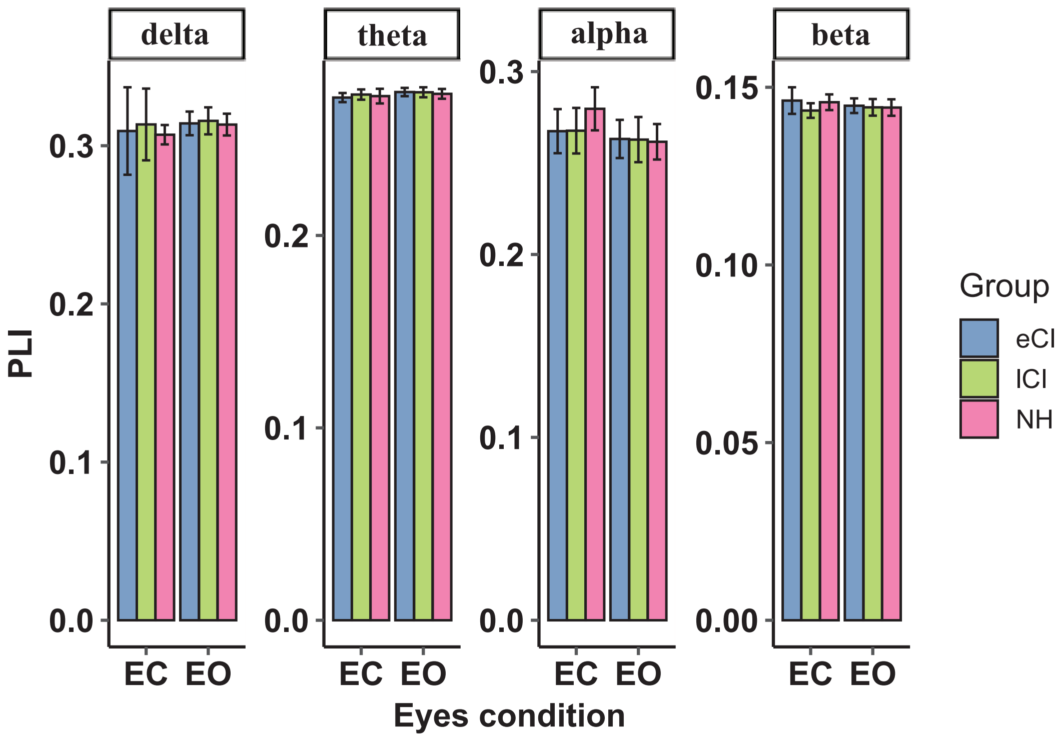

Figure S2. Functional connectivity between the left temporal cortex and left parietal cortex. The average functional connectivity between the left temporal and left parietal region was defined by the phase lag index (PLI) (y-axis) under the eyes-closed (EC) and eyes-open (EO) conditions (x-axis) in delta, theta, alpha, and beta frequency bands (top) in children with early cochlear implantation (eCI), children with late cochlear implantation (lCI), and children with normal hearing (NH). There were no statistically significant group differences for any of the frequency bands.

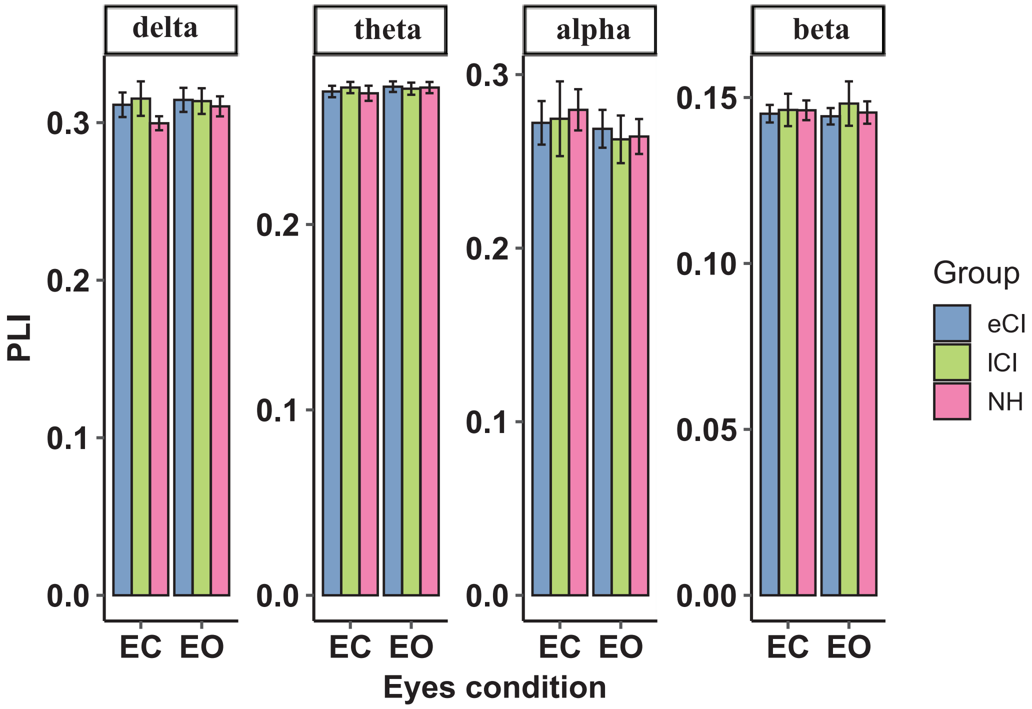

Figure S3. Functional connectivity between the right temporal cortex and right parietal cortex. The average functional connectivity between the right temporal and right parietal region was defined by the phase lag index (PLI) (y-axis) under the eyes-closed (EC) and eyes-open (EO) conditions (x-axis) in delta, theta, alpha, and beta frequency bands (top) in children with early cochlear implantation (eCI), children with late cochlear implantation (lCI), and children with normal hearing (NH). There were no statistically significant group differences for any of the frequency bands.
